# The estimated 10-year risk of first-onset cardiovascular disease in Swedish-born and non-Swedish-born primary healthcare patients

**DOI:** 10.1186/s12875-024-02446-w

**Published:** 2024-06-04

**Authors:** Mustafa Saleh, Helena Salminen, Marina Taloyan

**Affiliations:** 1 Medical programme, Karolinska Institutet, Stockholm, Sweden; 2 Division of Family Medicine and Primary Care, Department of Neurobiology, Care Sciences and Society (NVS), Karolinska Institutet, Alfred Nobels allé 23, Stockholm, SE-14183 Sweden; 3 Academic Primary Health Care Centre, Stockholm County Council, Stockholm, Sweden

**Keywords:** SCORE2, Cardiovascular risk assessment, Sweden, Immigrants

## Abstract

**Background:**

SCORE2 has been introduced as an updated risk assessment tool for calculating the 10-year risk of first-onset cardiovascular disease (CVD). However, it does not account for ethnicity or socioeconomic status, known to affect CVD risk. This study investigated and compared SCORE2 estimates in Swedish-born and non-Swedish-born primary healthcare patients. The second aim was to examine if several risk factors could explain differences in CVD risk between the groups.

**Methods:**

This was an observational, cross-sectional study. Data were obtained from the 4D Diabetes Project study, providing a total of 444 participants aged between 40 and 69 years. All participants had complete risk variable data necessary for the SCORE2 tool and no history of previous CVD. Descriptive analysis was conducted to compare distributions of risk factors between Swedes and immigrants and odds ratios of risk factors amongst these two groups in correlation to elevated CVD risk were calculated using logistic regression.

**Results:**

Swedish-born patients showed a significantly higher risk of elevated CVD risk estimates (≥ 2.5% CVD risk increase for individuals < 50 years, respectively, ≥ 5% for individuals aged 51–69) than the non-Swedish-born population, even after adjustment for educational level (OR = 1.61, 95% CI 1.08–2.39). Weekly alcohol consumption implicated a risk of being classified as high risk of CVD risk, regardless of country of birth (OR = 1.93 CI 1.25-3.00). However, Swedes accounted for most of the alcohol consumption (62.6% vs. 19.6%). No other explanatory variable showed significance in association with elevated CVD risk.

**Conclusions:**

Swedish-born patients were found to be at higher risk of an increased 10-year CVD risk. The association of alcohol consumption with elevated CVD risk needs to be further studied in longitudinal studies in representative populations, notably among Sweden’s diverse ethnic groups.

## Background

Cardiovascular diseases (CVDs) are currently the most common fatal non-communicable diseases and the leading cause of premature mortality worldwide, accounting for nearly one-third of all global deaths, with approximately 17.9 million deaths in 2019 [1]. The mortality from CVD is similar in Sweden with CVD-related deaths being overrepresented among men [2]. However, the number of deaths from CVD has been steadily decreasing during the last decades, thanks to general improvements in public health, changes in lifestyle, and continuous advancements in the field of cardiological care [2, 3]. Statistics from the National Board of Health and Welfare of Sweden show that “diseases of the circulatory system”, which includes CVDs, accounted for just over 28% of total deaths in 2020, a decrease of over 6% since 2016.

Key risk factors for Myocardial Infarction (MI) and stroke include hypertension, smoking, and elevated lipids [4–6]. Furthermore, several epidemiological and case-control studies have provided evidence of an association between lower alcohol intake and lower risks of CVD [7, 8].

The European Society of Cardiology (ESC) broadened guidelines in 2003 to cover all CVDs, introducing the Systematic Coronary Risk Evaluation (SCORE) project [9]. SCORE, widely used in Sweden, estimates the 10-year CVD mortality risk based on sex, smoking, systolic blood pressure (SBP), total cholesterol, and age as exposure time to risk [9]. The 2003 SCORE project yielded two age- and sex-adjusted risk charts, with a national adaptation for Sweden, introduced in 2004 [10]. Updated in 2015 using the MONICA survey data, the revised SCORE provided a more accurate risk estimation by Karjalainen et al. in 2017 [11]. However, SCORE focused on fatal event risks, potentially underestimating total cardiovascular risk in younger populations, and not accounting for competing risks, which may overestimate CVD risk in older individuals [12]. This results in a tendency to over-treat older individuals with a moderate CVD risk and high risk of other fatal outcomes while failing to prevent CVD outcomes in younger individuals who could benefit from interventions [12]. In 2021, SCORE2 was developed, addressing previous models’ limitations by estimating both non-fatal and fatal CVD risks and considering competing risks. This model uses non-HDL cholesterol for better accuracy and is recalibrated with up-to-date statistics, offering four risk charts based on national CVD mortality rates [13–15]. For diabetics, specific calculators from the Swedish National diabetes registry are recommended [13, 14, 16].

Sweden has been classified as a moderate-risk country based on age- and sex-standardized overall CVD mortality of 109 per 100,000 according to the most recent WHO statistics, whereas low-risk countries had < 100 deaths per 100,000 people. Therefore, the moderate risk chart has been introduced in Sweden [17].

The 2021 ESC guidelines on CVD prevention in clinical practice have suggested the classification of SCORE2 risk percentage in three different risk categories (low to moderate, high, and very high) with dissimilar cut-off levels by age. For instance, a low to moderate 10-year CVD risk is classified as < 2.5% in individuals younger than 50 years, respectively < 5% in individuals aged between 51 and 69 years. Lifestyle changes and medical interventions should be considered for individuals at high risk and are generally recommended for individuals at very high risk [16]. One limitation of the SCORE2 model was the inaccessibility to analyze data on several potentially confounding factors since they could not be obtained in the cohorts used for the model derivation. These include ethnicity, socioeconomic status (SES), nutrition, and physical activity among others [18].

Currently, little is known about the disparities between the Swedish-born and non-Swedish-born population’s risk of CVD mortality and morbidity. A few studies have examined the association between CVD and CHD morbidity and birthplace in Sweden. One follow-up study was conducted by Gadd et al. using incidence data from the Swedish MigMed database between 1997 and 1998, including over 3.5 million individuals aged between 35 and 64 years, where 550,000 were foreign-born. The study showed that the incidence rate of CVD/CHD was higher in most non-Swedish-born groups for both sexes, particularly individuals born in Finland, Poland, and the Middle East, while the Swedish-born population had incidence rates close to the median, even when considering SES It was suggested that the increased risk in these groups could be of a single- or multi-factorial origin such as lifestyle factors, smoking, etc. However, they were unable to take risk factors like blood pressure, lipids, and smoking habits into consideration because of the lack of data in the MigMed register. Therefore, they concluded that further studies regarding these risk factors were needed to undo the origin of these disparities [19]. In a recently published study, the Framingham Risk Score was applied to data from 830 participants in the *4D Diabetes Project* to investigate the correlation between CVD risk and place of birth. The population sample was dichotomized into a Swedish-born and foreign-born group, with most of the latter originating from the Middle East. Contrary to previous research, the findings showed that participants born in Sweden had a higher 10-year risk of CVD compared to those born abroad [20].

By implementing the SCORE2 prediction model in a population group with a high demographic of non-Swedish-born individuals, it could be possible to provide contemporary statistics, representing possible differences between the Swedish-born and non-Swedish-born population regarding the 10-year risk of fatal and non-fatal CVD, and further, to examine influencing county-level factors associated with the CVD morbidity and mortality rates in these groups. Clinically, this would help determine if physicians in primary health care should approach the country of birth as a relevant risk factor and better streamline the identification of patients at risk.

The primary aim of this study was to examine and compare the 10-year risk of first-onset CVD events in Swedish-born and non-Swedish-born primary healthcare patients using SCORE2. A secondary aim was to examine and compare risk factors that could explain eventual differences in CVD risk between the groups. The hypothesis was that the foreign-born group would show an increased 10-year risk of CVD in comparison to the Swedish-born group.

## Methods

This cross-sectional study was based on material collected for a pilot study called the 4D diabetes project (4DDP) as part of the 4D program, which was a collaboration between Karolinska Institutet and the Stockholm County Council. The 4D program focused on 4 common diagnoses in Sweden: arthritis, breast cancer, diabetes, and heart failure. The two main aims of the 4DDP were to promote early identification of prediabetes and diabetes using screening methods and to establish a biobank for future research. The data for the 4DDP was collected between 2013 and 2015 at two Academic Primary Health Care Centres (APHCCs): Flemingsberg and Jakobsberg. The 4DDP project was pilot study without estimation of statistical power. The centres were strategically selected due to their over-representation of immigrant patients, born in regions where the population has been identified as having an increased risk of developing diabetes. The participants were recruited at the APHCC waiting rooms where they received verbal and written information explaining the pilot study with access to translation in Swedish, English, Turkish, Arabic, or Persian. Additionally, an interpreter was available if needed. Patients who were interested and met the inclusion criteria had to provide informed consent and received a questionnaire mainly based on FINDRISK, a questionnaire model used to calculate the predicted risk of developing diabetes within 10 years. The questionnaire included questions regarding age, BMI, alcohol consumption, daily physical activity, daily vegetable and/or fruit intake, and medication use. A set of complementary questions were included as well regarding country of birth, smoking habits, and self-rated health. Furthermore, data on previous diagnoses and medications were obtained from the patient’s journals with their permission. The patients had to submit venous blood samples at Karolinska University Hospital. Total cholesterol, as well as HDL cholesterol, were analyzed as part of the venous blood samples. Additionally, participants in the study underwent a physical exam at the APHCC that included blood pressure measurements.

**Fig. 1:**
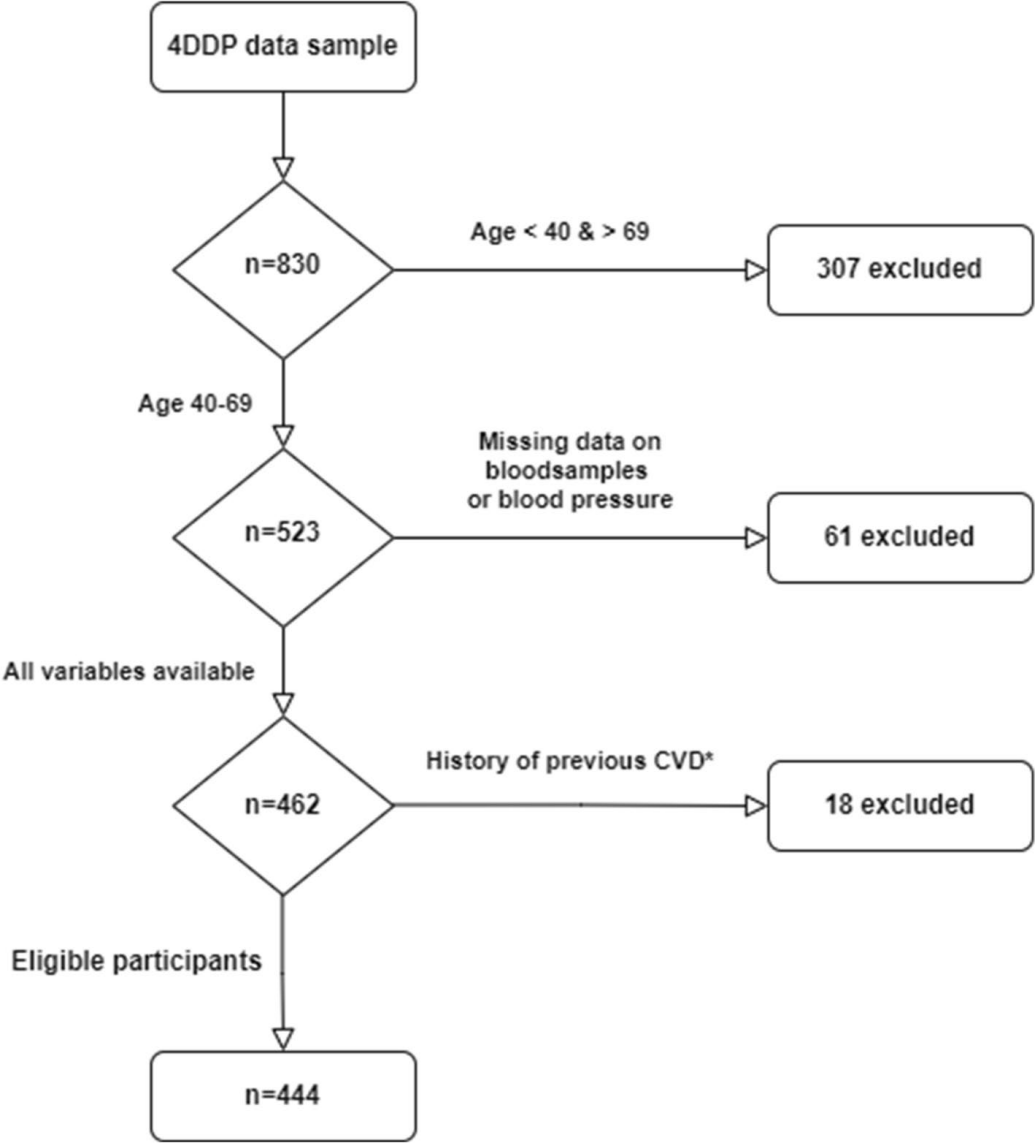
Flowchart of selection of study population. Abbreviations 4DDP, 4D diabetes project

A total of 830 patients were included in the 4DDP’s pilot study between the ages of 18 to 74 years. To be eligible to participate, they had to either be born in Sweden with Swedish-born parents or be born in Africa, the Middle East, or Asia, or have one or both parents from these regions. Previously diagnosed diabetes, severe psychiatric disorders, and pregnancy were set as exclusion criteria for the 4DDP. For this study were additional exclusion criteria set to ensure adequate and representative risk estimations, including lack of required variables (61 excluded), age < 40 and > 69 years (307 excluded), and a history of previous CVD (18 excluded), with the following available ICD-10 codes classified as CVDs; I20, I25, I48, I51, I63, and I69. This process yielded a total of 444 eligible participants, as demonstrated by the flowchart in Fig. 1.

The information on birthplace from the questionnaire was used to define the participant as Swedish-born or non-Swedish-born. The primary outcome of this study is the SCORE2 risk estimate categorized as either “low to moderate-” or “high-/very high-risk”. These risk groups were defined following the age-adjusted cut-off values recommended by the 2021 ESC guidelines. According to this guideline, “low to moderate” is defined as a 10-year CVD risk < 2.5% in individuals younger than 50 years, respectively, < 5% in individuals aged between 50 and 69 years. “High” is defined as a 10-year CVD risk of 2.5 − 7.5% for the former age group and 5 − 10% in the latter, while “very high” is defined as a 10-year CVD risk ≥ 7.5% for the former and ≥ 10% for the latter.

However, when conducting binary logistic regression for our analysis, we decided to merge the ‘high’ and ‘very high’ risk groups into a single category. This decision was made to create a simpler binary outcome, where individuals exceeding the ESC risk recommendations, whether ‘high’ or ‘very high,’ were included in one group. This binary categorization allowed for a more straightforward statistical analysis, ensuring that our model could effectively differentiate between those meeting the ESC risk recommendations and those exceeding them. This approach simplifies the interpretation of our logistic regression results and facilitates a clearer understanding of the impact of CVD risk on our study outcomes.

The descriptive data presents covariates required for the calculation of SCORE2 in the study population and by birthplace. This includes *age*, *sex, total cholesterol*, *HDL-cholesterol*, *SBP*, and *smoking*. Additional data retrieved from the original dataset included information regarding CVD preventive *medication use*, *alcohol consumption*, and *education*. Alcohol consumption was originally divided into five categories in the questionnaire but was dichotomized in this study. One group included those who reported " less than one standard glass unit per week or never” and the other group was formed by those who reported “1–4 units/week”, “5–9 units/week”, “10–15 units/week”, and “15 or more units/week”. Furthermore, since two values on SBP were available in the original dataset, the SBP used for calculation was set as the mean value of both observations. Binary variables were disclosed as frequencies and percentages and continuous variables were presented as mean ± standard deviation (SD). Variables with non-normally distributed data were described as the median and interquartile range (IQR).

### Statistical analysis

Statistical analysis was performed using STATA statistical software version 14.2. The SCORE2 algorithm calculation using STATA was acquired from the Cardiovascular Epidemiology Unit of the University of Cambridge [21]. SCORE2 covariates were tabulated by birthplace. Frequencies in studied groups and tests of significant differences between them were performed by the Chi-squared test (for normally distributed variables) and the Mann-Whitney’s test (non-normally distributed variables). By implementing the SCORE2 algorithm within STATA, an age- and sex-adjusted risk estimation was acquired for the moderate region risk model. SCORE2 risk estimates, attained in percentages, were further categorized into one of three risk category groups. The following explanatory variables were continuous: *age, total cholesterol, HDL*, and *systolic blood pressure*. *Sex, alcohol consumption, education, physical activity, BMI, fruits and vegetables*, and *red meat* were dichotomized variables.

Unconditional logistic regression was performed with “elevated SCORE2-risk” set as the outcome variable to obtain odds ratios and adjust for explanatory variables. Goodness-of-fit-test was used on the last model to confirm accuracy. *P*-values < 0.05 were considered significant.

## Results

### Participants’ characteristics and SCORE2 prevalence

The exclusion of ineligible participants resulted in a final study population of 444 patients with a final number of 51 countries of birth reported. The ratio of immigrants to Swedes was 3:2, with the majority originating in the Middle East, including 59 in Turkey, 28 in Iran, and 21 in Iraq. Most of the participants were women (56.8%) with a comparable distribution in both groups (Table 1). When compared to Swedish-born participants, the proportion of non-Swedish-born participants with less than 9 years of education as well as over 12 years of education was significantly higher (25.8% vs. 11.7% and 54.5 vs. 48.1%; *P* < 0.001) respectively. Mean SBP was significantly higher for Swedish-born participants 127.3 mmHg ± 17.1 vs. 122.0 mmHg ± 16.6, respectively (*P* = 0.001). Conversely, a significantly higher percentage of the non-Swedish-born group were smokers (23.4% vs. 14.0%; *P* = 0.01).

**Table 1:** SCORE2 variable and participant characteristics by birthplace, *n* = 444

Risk factor characteristics	Total Population (*N* = 444) mean ± SD or *n* (%)		Swedish-born (*n* = 179) mean ± SD or *n* (%)	Non-Swedish-born (*n* = 265) mean ± SD or *n* (%)	*P*-value
**Age**, ***years***, [range]	54 [47–61]		57 [50–64]	52 [46–59]	**< 0.001** ^**a**^
**Sex**					0.75^b^
Male	192 (43.2)		79 (44.1)	113 (42.6)	
Female	252 (56.8)		100 (55.9)	152 (57.4)	
**Total Cholesterol**, *mmol/L*	5.2 ± 1.0		5.3 ± 1.1	5.1 ± 1.0	0.07^c^
**HDL**, *mmol/L*	1.4 ± 0.4		1.5 ± 0.4	1.4 ± 0.4	**0.001** ^c^
**Systolic BP**, *Mm Hg*	124.2 ± 16.9		127.3 ± 17.1	122.0 ± 16.6	**0.001** ^c^
**Current smoker**, n (%)	87 (19.6)		25 (14.0)	62 (23.4)	**0.01** ^**b**^
**Alcohol**, *weekly consumption*					**< 0.001** ^**b**^
No	280 (63)		67 (37.4)	213 (80.4)	
Yes	164 (37)		112 (62.6)	52 (19.6)	
**Education**, *years*					**< 0.001** ^**b**^
< 9	89 (20.1)		21 (11.7)	68 (25.8)	
> 9 to 12	124 (28.0)		72 (40.2)	52 (19.7)	
> 12	230 (51.9)		86 (48.1)	144 (54.5)	
**Physical activity**, *minutes/week*					**< 0.001** ^**b**^
< 150	214 (48.2)		53 (29.6)	161 (60.7)	
≥ 150	230 (51.8)		126 (70.4)	104 (39.3)	
**BMI**, *kg/m*^*2*^					**0.04** ^**b**^
NW < 25	86 (19.4)		45 (25.1)	41 (15.4)	
OW 25-29.9	186 (41.9)		70 (39.1)	116 (43.8)	
OB ≥ 30	172 (38.7)		64 (35.8)	108 (40.8)	
**Fruits & vegetables**, *daily intake of both*					0.72^**b**^
No	203 (45.7)		80 (44.7)	123 (46.4)	
Yes	241 (54.3)		99 (55.3)	142 (53.6)	
**Red meat**, *weekly consumption*					**0.03** ^**b**^
Rarely^*^	87 (19.6)		26 (14.5)	61 (23.0)	
Frequently^**^	357 (80.4)		153 (85.5)	204 (77.0)	

Statistically significant *P*-values are marked in bold

*Abbreviations:HDL* High-density lipoprotein, *BP* Blood pressure, *SD* Standard deviation, *IQR* Interquartile range, *NW* Normal weight, *OW* Overweight, *OB* Obese

^a^*P*-value determined by Mann-Whitney’s test

^b^*P*-value determined by Chi-squared test

^c^*P*-value determined by T-test

^*^1 meal/week or less. ^**^ Several meals/weeks to daily consumption

### Use of statins and hypertensive medication

Although this study excluded patients with previous CVDs, there was still data on current medication with statins for some of the patients within the total study population (12.6%) as well as the use of antihypertensive drugs (22.8%). There was, however, no significant difference in medication uses between the Swedish-born and non-Swedish-born groups. It is unknown if these patients received medications as primary or secondary prevention.

### Risk estimates by birthplace

**Fig. 2:**
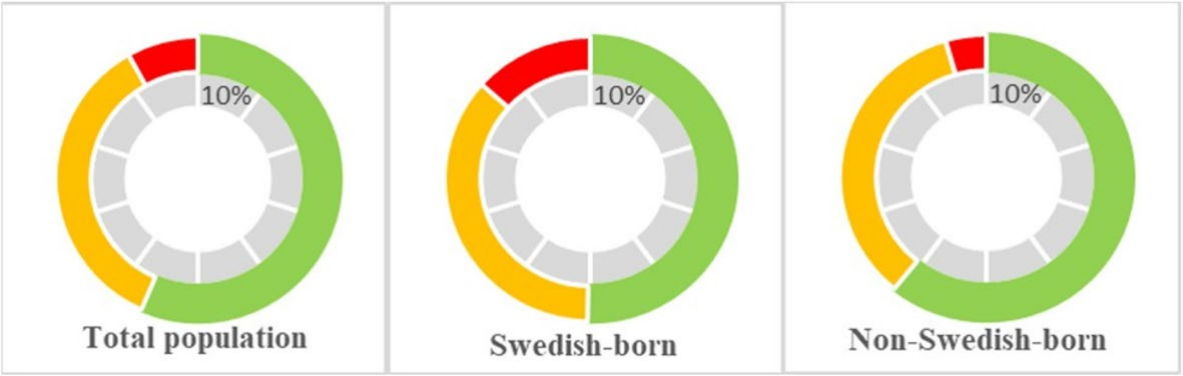
Visualizing circle diagrams of CVD-risk estimates for all groups and the total population. The colors indicate the first three risk categories presented in Table 2: low-to-moderate, high, and very high risk

Of all 444 participants, 193 (43.5%) had a high or very-high 10-year risk of first-onset CVD, based on a risk percentage > 2,5% for ages 40–49 and > 5% for ages 50–69 (Table 2; Fig. 2). The proportion of Swedish-born patients categorized as high-risk was significantly higher than the counterpart group (36.3% vs. 34.7%; *P* = 0.002). In the very-high-risk category, the proportion of Swedish-born patients was, yet again, significantly higher than in the non-Swedish-born patients (13.4% vs. 4.5%; *P* = 0.002).

**Table 2:** SCORE2 risk category outcome by birthplace, *n* = 444

SCORE2 risk categories	Total Population (*n* = 444) *n* (%)	Swedish-born (*n* = 179) *n* (%)	Non-Swedish-born (*n* = 265) *n* (%)	*P*- Value^b^
Low to Moderate	251 (56.5)	90 (50.3)	161 (60.8)	0.03
High	157 (35.4)	65 (36.3)	92 (34.7)	**0.002**
Very High	36 (8.1)	24 (13.4)	12 (4.5)	**0.002**
High to Very High^a^	193 (43.5)	89 (49.7)	104 (39.2)	**0.03**

^a^ Demonstrates all participants over the recommended age-adjusted risk

^b^*P*-value determined by Chi-squared test

### Risk estimates in male participants by birthplace

There was a significant difference when solely comparing male participants from both groups in the very-high-risk category (Table 3). A lower part of non-Swedish-born participants was found to be at very high risk in comparison to the Swedish-born participants (9.7% vs. 22.8%; *P* = 0.03). However, no significant difference was found between the groups regarding the other risk categories.

**Table 3:** SCORE2 risk category outcome for male participants by birthplace, *n* = 192

SCORE2 risk categories Male sex	Swedish-born (*n* = 79) *n* (%)	Non-Swedish-born (*n* = 113) n (%)	*P*-value
**Low to Moderate**	25 (31.6)	42 (37.2)	0.43^a^
**High**	36 (45.6)	60 (53.1)	0.30^a^
**Very High**	18 (22.8)	11 (9.7)	**0.03** ^**a**^

^a^*P*-value determined by Chi-squared test

### Analyzing odds ratios for abnormal CVD risk by birthplace

As a significant difference was found between the Swedish-born and non-Swedish-born participants’ SCORE2 risk estimates, unconditional logistical regression was performed to estimate the odd ratios for having a high-risk or very-high-risk SCORE2 estimate in both groups, as well as stepwise adjustment for explanatory variables that are not integrated into the SCORE2 algorithm. Accordingly, three models were formed where educational level is added in model 2 and alcohol in model 3 (Table 4). Other variables did not show any significance and were therefore not included. Since the SCORE2 algorithm already includes adjustment for sex and age, there was no further adjustment for these variables in the logistic regression. A goodness of fit test at 0.934 for model 3 indicates that the final model had a good fit.

**Table 4:** The odds ratios (OR) with 95% confidence intervals (95% CIs) of risk factors for an elevated 10-year risk of CVD^a^ in the Swedish-born group with the non-Swedish-born group as a reference

Variables	Model 1	Model 2	Model 3
**Non-Swedish-born Swedish-born**	1 **1.53 (1.04–2.25)**	1 **1.61 (1.08–2.39)**	1 1.21 (0.78–1.88)
**Education**, *years* **< 9** **> 9 to 12** **> 12**		1 0.70 (0.40–1.23) 0.71 (0.43–1.18)	1 0.69 (0.39–1.22) 0.66 (0.40–1.09)
**Alcohol**, *weekly consumption* **No** **Yes**			1 **1.93 (1.25-3.00)**

^a^Elevated 10-year risk of CVD defined as high or very-high SCORE2 category. Statistically significant *P*-values are marked in bold

The crude odds ratio for having a risk that exceeds the ESC risk recommendations was 1.53 times higher (95% CL = 1.04–2.25) in the Swedish-born group compared to the non-Swedish-born group. A slight increase is shown when adjusting for education in model 2. However, no significance was found between the educational level groups. After the adjustment for alcohol, the OR of birthplace decreases and loses its significance. Additionally, those who had a weekly consumption of alcohol had almost twice higher odds (OR = 1.93,95% CL = 1.25-3.00) of having an elevated 10-year CVD risk than those who did not regardless of birthplace.

## Discussion

In this cross-sectional study, we examined disparities regarding the estimated 10-year risk of fatal and non-fatal CVD events between Swedish-born and non-Swedish-born primary healthcare patients, using the SCORE2 model. We also examined if several additional risk factors could explain the differences between the groups. We hypothesized that non-Swedish-born patients would have a higher 10-year risk of CVD in comparison to Swedish-born patients.

The findings showed that Swedish-born patients had a significantly higher frequency of elevated CVD risk in this population, particularly in the very-high-risk group. They also showed around 60% higher risk of exceeding the ESC recommendation than the non-Swedish-born patients when adjusting for education. None of the explanatory variables showed any significance in association with elevated CVD risk and birthplace, except for alcohol consumption.

Alcohol consumption was found to be a significant confounder since adjustment for alcohol consumption caused birthplace to lose significance while showing a significant association with elevated CVD risk. Participants who admitted to weekly alcohol consumption had twice the increased CVD risk regardless of birth country. However, the Swedish-born population accounted for most of the alcohol consumption (62.6% vs. 19.6%) which explains the association with birthplace. Additionally, there was a significant association between alcohol consumption and SBP with a higher mean value among participants who admitted to weekly alcohol consumption and a low mean value for those who denied weekly consumption (*P* = < 0.001). These findings suggest that alcohol consumption alone could be part of the explanation of the disparities between the Swedish-born and non-Swedish-born groups, possibly by affecting SBP and, subsequently the SCORE2 estimate.

Several previous meta-analytic studies investigating the association between alcohol and CV mortality have found the relationship to be “J-shaped”, i.e., alcohol decreases the risk in low-moderate consumption and increases the risk in high consumption [22, 23]. However, several cohort studies have been criticized for methodological biases such as the classification of former heavy alcohol consumers as abstainers. A large study by Xi et al. that used lifetime abstainers as the reference group to address this bias while also adjusting for cofounders came to a similar conclusion that light to moderate drinking might decrease the risk of CVD-related mortality when compared to alcohol abstainers [24]. Our outcome depends on the SCORE2 model which differs from other cohort studies since it calculates the 10-year risk of both fatal and non-fatal CVD events. Since information about alcohol habits in our study was limited by the questionnaire in the pilot study, we could not exclude occasional drinkers or former heavy alcohol consumers from the group who admitted to no weekly alcohol consumption, which might have contributed to a bias in these findings. Furthermore, we stratified the variable into two groups reflecting proportional distributions, with a noteworthy 63% of respondents indicating a consumption level of “less than 1 standard unit or never.” The markedly diminished response rates for the remaining three categories may exert a consequential influence on the study’s findings. It is nevertheless clear that weekly alcohol intake was associated with an increased SCORE2 estimate.

In the follow-up study conducted by Gadd et al., they analyzed CVD and CHD incidents from three and a half million people in Sweden aged 35–64 from 13 countries of birth who were defined and compared to a Swedish-born group. The incidence rates and relative risk of CVD and CHD were found to be higher in most non-Swedish-born groups for both sexes, even when adjusting for educational level and employment status. This included people born in Turkey, Iraq, Finland, and Poland among others [19]. Our findings are contrary to those of Gadd et al. since the Swedish-born group was found to be at a higher 10-year risk of first-onset CVD events than the non-Swedish-born group, even when adjusting for educational level. There are, however, several methodological differences that might have influenced the contradictory results. Firstly, a major difference is the use of SCORE2 in our study to calculate the risk of future CVD events instead of analyzing incidence rates. Secondly, Gadd et al. were limited by the data in the MigMed registry and were not able to consider explanatory variables, whereas our study enabled the use of risk factors in the SCORE2 calculation as well as an analysis of the association between explanatory variables (that showed significant differences between the groups) and increased CVD-risk. Thirdly, there was an immense difference in sample size. The fact that our study included 265 non-Swedish-born participants from 51 different countries of origin limited us from splitting them into smaller groups, which might have influenced our results since Gadd et al. reported lower incidence rates of CVD in several countries.

Although several dissimilarities were found between Gadd et al.‘s study and ours, this study shares Gadd et al.‘s conclusion that men were found to be at significantly higher risk of CVD in both Swedish-born and non-Swedish-born groups, compared to women.

Furthermore, our study shares the findings presented by the recently published Framingham study [20]. Additionally, both studies (Framingham and the current) found alcohol consumption to be significantly associated with an increased CVD risk in the study population. Although the same data set was used for both studies, our study utilized dissimilar inclusion and exclusion criteria due to the different risk estimation models used, nearly halving our study population. Finally, our findings add credibility to the conclusion of Taloyan et al. since the use of two different established risk estimation tools has come to similar conclusions. However, which risk score estimate to favor when addressing the foreign-born Swedish population has yet to be determined.

### Clinical implications

Mapping out disparities in the population is an important aid to achieving the goal of health equity. By the identification of groups at risk, we can engage patients with, for example, socioeconomic or ethnic disadvantages in primary health care. Our findings showed an association between alcohol consumption and elevated SCORE2 estimates. This emphasizes the importance of addressing the increased consumption of alcohol in primary care as a potential risk factor for CVD. Although the risk was found to be similar regardless of birthplace, alcohol consumption is more frequent in some socioeconomic groups and ethnicities. Therefore, targeting these groups and engaging in prophylactic measures could help reduce the difference in risk between Swedes and immigrants as well as the overall risk of CVD in the population.

### Strengths and limitations

Few studies so far have investigated disparities associated with ethnicity when it comes to CVD morbidity and mortality in the Swedish population. Our study seems to be the first to use a well-established risk estimate model to examine differences in risk and account for explanatory variables instead of only looking at incidence rates. This methodological approach is a major strength of this study as it allows for the identification of risk factors associated with increased CVD risk. Additionally, the Academic Primary Healthcare Centres from which the study participants were invited (Flemingsberg and Jakobsberg) were well chosen since both regions have an over-representation of immigrants, which enabled the comparison of Swedish-born and non-Swedish-born individuals in our study. On the other hand, our study was limited by the lack of data concerning individuals’ migration status and the duration of their resettlement in Sweden, preventing an in-depth analysis of “health migration” phenomena [25].

However, there was no record of the number of patients who declined participation and their reason for decline which increases the risk of non-response bias. Another weakness of this study is the grouping of all ethnicities other than those who were Swedish-born as the non-Swedish-born group since previous studies discovered differences in incidence rates between countries. Moreover, the data retrieved from the pilot study regarding lifestyle factors were not collected specifically for this study’s aim, resulting in suboptimal categorizing of alcohol consumption which prevented us from adjusting for biases presented by previous studies. Furthermore, as previously stated, there is a well-established connection between SES and CVD morbidity. To address this, we adjusted our results for the educational level, however, further inclusion of employment status when adjusting for SES might have increased the legitimacy of our results.

The limitations highlight areas for further research. Due to the limitation in sample size and the large number of countries reported in this study, we divided the study population into two groups. This might cause bias due to disparities within the non-Swedish-born group. Future studies should address this bias by limiting the comparison group to a specific birth country, or region with acknowledged similarities in CVD risk, to contribute to a better understanding of ethnicity’s role as a risk factor when confronting CVD risk.

Moreover, accurate adjustment for explanatory variables obtained from questionnaires relies on the use of valid and reliable questionnaires [26]. Using questions with cut-off values in concordance with contemporary studies could benefit the overall quality of future studies and open opportunities to adjust for biases such as the abstainers-bias presented in the earlier mentioned study by Xi et al. [24]. Finally, SCORE2-OP was not used in our study due to the lack of participants over 69 years. Future researchers in this field should consider comparing older individuals by using the risk model mentioned above, to provide better coverage of the whole population. Additionally, due to SCORE2 not being applicable for individuals with type 2 diabetes, we excluded these patients from our study, which might limit the broader applicability of our results. Given that many people with type 2 diabetes have various risk factors for cardiovascular disease (CVD), future research would benefit from including this group to ensure more comprehensive findings.

### Conclusions

This study concludes that the likelihood of having an increased 10-year risk of first-onset CVD was higher for Swedish-born patients compared to non-Swedish-born patients. The association of alcohol consumption with elevated CVD risk needs to be further studied in longitudinal studies in representative populations, notably among Sweden’s diverse ethnic groups.

## Data Availability

Data and materials are available to be shared from the last author (MT) on reasonable request.

## Abbreviations

APHCC: Academic primary health care centre
CHD: Coronary heart disease
CVD: Cardiovascular disease
DALYs: Disability-adjusted life years
IHD: Ischemic heart disease
IQR: Interquartile range
MI: Myocardial infarction
SCORE: Systematic coronary risk evaluation
SES: Socioeconomic status
SD: Standard deviation
WHO: World health organization
